# Supplementary material for: Realistic nitrate concentrations diminish reproductive indicators in Skiffia lermae, an endemic species in critical endangered status
Source: PeerJ. 2024 Sep 9;12:e17876. doi: 10.7717/peerj.17876 (PMC11391940; doi:10.7717/peerj.17876)
Supplement: Supplemental Information 1 [file peerj-12-17876-s001.docx]

**Supplementary Table 1. Developmental stages of oocytes in the female gonads of viviparous fish.**

| **Gonadal stage** | **Characteristics** |
| --- | --- |
| Stage I | Early primary growing oocytes with single or multiple nucleoli |
| Stage II | Medium primary growth oocytes, characterized by the presence of Balbiani bodies |
| Stage II | Primary growth oocytes, characterized by the appearance of large oil droplets |
| Stage IV | Primary secondary growth oocytes, characterized by the appearance of the first yolk globules |
| Stage V | Late growing oocytes, yolk droplets coalesce and form a large yolk globule which occupies most of the volume of the oocyte; at this time oil droplets are at the periphery of the ooplasm |
| Stage VI | Fully developed oocytes, at this stage females are ready to mate |

(Uribe et al., 2012; Tinguely et al., 2019).
