# Supplementary material for: Realistic nitrate concentrations diminish reproductive indicators in Skiffia lermae, an endemic species in critical endangered status
Source: PeerJ. 2024 Sep 9;12:e17876. doi: 10.7717/peerj.17876 (PMC11391940; doi:10.7717/peerj.17876)
Supplement: Supplemental Information 2 [file peerj-12-17876-s002.docx]

**Supplementary Table 2. Characteristics and classification of atretic oocytes.**

| **Stages of atresia** | **Characteristics** |
| --- | --- |
| Alpha stage | Characterized by alterations in the ovoplasm, proliferation of follicular cells and ruptures in the zona pellucida |
| Beta stage | Characterized by ovoplasm reduction due to diffusion and phagocytosis by follicular cells |
| Gamma stage | Characterized by follicular cells and vascularized theca occupying the oocyte |
| Delta stage | Characterized by a progressive decrease in the volume of the atretic follicle |

(Uribe et al. 2006).
