# Supplementary material for: Realistic nitrate concentrations diminish reproductive indicators in Skiffia lermae, an endemic species in critical endangered status
Source: PeerJ. 2024 Sep 9;12:e17876. doi: 10.7717/peerj.17876 (PMC11391940; doi:10.7717/peerj.17876)
Supplement: Supplemental Information 3 [file peerj-12-17876-s003.docx]

**Supplementary Table 3. Summary of the best generalized linear mixed models explaining latency for food detection, number of fish actively responsive to food and aquatic surface respiration in *Skiffia lermae.***

| **Response variable** | **Explanatory variables** | **Estimate (Standard error)** | **t value** | **Pr(>\|z\|)** |
| --- | --- | --- | --- | --- |
| **Latency for food detection** | Intercept  Concentration 5 mg/L  Concentration 10 mg/L  Concentration 20 mg/L  Second period  Third period  Concentration 5 mg/L: Second period  Concentration 10 mg/L: Second period  Concentration 20 mg/L: Second period  Concentration 5 mg/L: Third period  Concentration 10 mg/L: Third period  Concentration 20 mg/L: Third period | 2.647 (0.139)  0.081 (0.141)  -0.048 (0.141)  0.287 (0.141)  -1.699 (0.133)  -2.120 (0.133)  -0.267 (0.189)    0.131 (0.189)  0.630 (0.189)    0.387 (0.189)    0.390 (0.189)    0.377 (0.189) | 18.945  0.576  -0.346  2.038  -12.717  -15.872  -1.416    0.697  3.321    2.042    2.065    1.997 | **<0.001**  0.564  0.729  **0.041**  **<0.001**  **<0.001**  0.156    0.485  **<0.001**    **0.041**    **0.038**    **0.045** |
| **Number of fish actively responsive to food** | Intercept  Concentration 5 mg/L  Concentration 10 mg/L  Concentration 20 mg/L  Second period  Third period | 2.061 (0.034)  0.019 (0.036)  -0.007 (0.036)  -0.033 (0.037)  0.223 (0.034)  0.243 (0.033) | 59.144  0.530  -0.202  -0.907  6.570  7.171 | **<0.001**  0.596  0.840  0.365  **<0.001**  **<0.001** |
| **Aquatic surface respiration** | Intercept  Concentration 5 mg/L  Concentration 10 mg/L  Concentration 20 mg/L  Second period  Third period | 0.192 (0.136)  0.161 (0.076)  0.238 (0.074)  0.255 (0.074)  0.439 (0.075)  0.785 (0.071) | 1.415  2.124  3.190  3.432  5.805  10.934 | **0.007**  **0.033**  **0.001**  **0.001**  **<0.001**  **<0.001** |

Bold values denote significant effects at p ≤ 0.05.
