# Supplementary material for: Realistic nitrate concentrations diminish reproductive indicators in Skiffia lermae, an endemic species in critical endangered status
Source: PeerJ. 2024 Sep 9;12:e17876. doi: 10.7717/peerj.17876 (PMC11391940; doi:10.7717/peerj.17876)
Supplement: Supplemental Information 4 [file peerj-12-17876-s004.docx]

**Supplementary Table 4. Summary of the best generalized linear mixed models explaining absolute growth, specific growth rate and scaled mass index in *Skiffia lermae.***

| **Response variable** | **Explanatory variables** | **Estimate (Standard error)** | **t value** | **Pr(>\|z\|)** |
| --- | --- | --- | --- | --- |
| **Absolute growth** | Intercept  Concentration 5 mg/L  Concentration 10 mg/L  Concentration 20 mg/L  SexMale | 0.191 (0.015)  -0.008 (0.020)  -0.0003 (0.020)  0.009 (0.020)  0.008 (0.014) | 11.984  -0.399  0.017  0.494  0.566 | **<0.001**  0.690  0.987  0.621  0.572 |
| **Specific growth rate** | Intercept  Concentration 5 mg/L  Concentration 10 mg/L  Concentration 20 mg/L  SexMale | 3.376 (0.147)  -0.308 (0.186)  -0.071 (0.186)  -0.009 (0.186)  0.100 (0.131) | 22.911  -1.652  -0.385  0.050  0.764 | **<0.001**  0.0984  0.700  0.960  0.444 |
| **Scaled mass index** | Intercept  Concentration 5 mg/L  Concentration 10 mg/L  Concentration 20 mg/L  SexMale | 0.218 (0.005)  -0.009 (0.007)  0.002 (0.007)  0.005 (0.007)  0.007 (0.005) | 37.186  -1.255  0.391  0.757  1.463 | **<0.001**  0.210  0.696  0.449  0.143 |

Bold values denote significant effects at p ≤ 0.05.
