# Supplementary material for: Realistic nitrate concentrations diminish reproductive indicators in Skiffia lermae, an endemic species in critical endangered status
Source: PeerJ. 2024 Sep 9;12:e17876. doi: 10.7717/peerj.17876 (PMC11391940; doi:10.7717/peerj.17876)
Supplement: Supplemental Information 5 [file peerj-12-17876-s005.docx]

**Supplementary Table 5.** **Summary of the best generalized linear mixed models explaining immature red blood cells, the proportion of total epithelia available for gas exchange (PAGE_Tot_) index, liver damage tissue index (LDTI), density of stage III oocytes and percentage of atresia in *Skiffia lermae*.**

| **Response variable** | **Explanatory variables** | **Estimate (Standard Error)** | **t value** | **Pr(>\|z\|)** |
| --- | --- | --- | --- | --- |
| **Immature red blood cells** | Intercept  Concentration 5 mg/L  Concentration 10 mg/L  Concentration 20 mg/L  SexMale | 64.900 (9.075)  -0.100 (11.480)  4.800 (11.482)  5.700 (11.481)  10.800 (8.115) | 7.152  -0.009  0.418  0.496  1.331 | **<0.001**  0.993  0.676  0.620  0.183 |
| **PAGE_Tot_ index** | Intercept  Concentration 5 mg/L  Concentration 10 mg/L  Concentration 20 mg/L  SexMale  Concentration 5 mg/L:  SexMale  Concentration 10 mg/L: SexMale  Concentration 20 mg/L: SexMale | -1.484 (0.044)  -0.196 (0.045)  -0.499 (0.047)  -0.757 (0.050)  0.144 (0.042)  -0.017 (0.062)  -0.166 (0.066)  0.346 (0.067) | -33.02  -4.35  -10.58  -15.02  3.39  -0.28  -2.50  5.15 | **<0.001**  **<0.001**  **<0.001**  **<0.001**  **<0.001**  0.776  **0.012**  **<0.001** |
| **LDTI** | Intercept  Concentration 5 mg/L  Concentration 10 mg/L  Concentration 20 mg/L  SexMale  Concentration 5 mg/L:  SexMale  Concentration 10 mg/L: SexMale  Concentration 20 mg/L: SexMale | 0.897 (0.047)  -0.150 (0.012)  -0.095 (0.013)  0.087 (0.014)  0.057 (0.014)  0.057 (0.019)  -0.008 (0.019)  -0.016 (0.021) | 18.825  -12.261  -7.186  6.020  4.012  2.954  -0.446  -0.759 | **<0.001**  **<0.001**  **<0.001**  **<0.001**  **<0.001**  0.003  0.648  0.447 |
| **Density of stage III oocytes** | Intercept  Concentration 5 mg/L  Concentration 10 mg/L  Concentration 20 mg/L | 0.031 (0.004)  0.011 (0.007)  0.023 (0.008)  0.034 (0.010) | 7.313  1.551  2.695  3.438 | **<0.001**  0.121  **0.007**  **<0.001** |
| **Percentage of atresia** | Intercept  Concentration 5 mg/L  Concentration 10 mg/L  Concentration 20 mg/L | -1.091 (0.387)  0.146 (0.495)  0.041 (0.508)  0.536 (0.516) | -2.817  0.296  0.082  -1.039 | **<0.001**  0.767  0.934  0.298 |

Bold values denote significant effects at p ≤ 0.05.
